# Using fungal–bacterial community analysis to explore potential microbiomes to manage *Meloidogyne incongnita*

**DOI:** 10.3389/fmicb.2024.1415700

**Published:** 2024-10-22

**Authors:** Qipeng Jiang, Yong Wang, Jiamin Yu, Jinfeng Wang, Shiping Guo, Dongyang Liu, Xiangwen Yu, Lianqiang Jiang, Gang Long, Daojiang Xi, Shuhong Chen, Yue Wang, Wei Ding

**Affiliations:** ^1^College of Plant Protection, Southwest University, Chongqing, China; ^2^Liangshan Branch of Sichuan Tobacco Company, Xichang, China; ^3^Sichuan Branch of China Tobacco Corporation, Chengdu, China

**Keywords:** root-knot nematodes, *Microbacterium*, fungal-bacterial interaction, tobacco resistance, co-occurrence network

## Abstract

Rhizosphere microbial communities strongly affect outbreaks of root-knot nematode (RKN) disease. However, little is known about the interactions among fungi, bacteria and RKN. The bacterial and fungal community compositions in the rhizospheres of four representative tobacco varieties, both resistant and susceptible to RKN, were characterized using 16S rRNA gene sequencing for bacteria and internal transcribed spacer gene sequencing for fungi. Our findings revealed that the fungi played crucial roles in facilitating the cross-kingdom and symbiotic fungal–bacterial interactions to suppress RKN. Moreover, our investigation suggested *Microbacterium* as a potential microbial antagonist against RKN based on its enhanced presence in RKN-resistant tobacco genotypes, and the relative abundance of *Microbacterium* was 34.49% greater in the rhizosphere of resistant tobacco than that of susceptible tobacco significantly. Notably, the richness of fungal community enhanced tobacco’s microbe-associated resistance to RKN through the positive regulation of the richness and diversity of bacterial community and the relative abundance of *Microbacterium*. This study underscores the critical role of the fungus–dominated fungal–bacterial community in bolstering tobacco resistance against RKN. The potential antagonistic role of *Microbacterium* presents promising avenues for innovative RKN management strategies.

## 1 Introduction

Root-knot nematodes (RKNs, *Meloidogyne* spp.) are the most destructive and widespread type of plant-parasitic nematode (PPN) and have been shown to significantly hinder nutrient and water uptake in plants, leading to developmental delays (Jones et al., 2013). RKN infection not only causes wounds in plant roots but also predisposes plants to further damage by other soil-borne pathogens, thereby leading to complex plant diseases (Kyndt et al., 2017; Zhang Y. et al., 2020). This has led to substantial economic losses globally, particularly affecting the production of *Solanaceae* crops, such as tomato (Du et al., 2022; El-Sappah et al., 2019), eggplant (Khan and Siddiqui, 2017; Zhou et al., 2019) and tobacco (Cao et al., 2023; Xu et al., 2023). In the context of the urgent demand for environmentally friendly strategies to manage RKNs, biocontrol is gaining increasing attention as a potential and sustainable strategy (Sun et al., 2021). This approach is recognized for maintaining the One Health of humans, soil, plants and animals (Berg et al., 2020).

The pivotal role of microbial communities associated with plants, often referred to as the ‘second genome’ of plants (Afzal et al., 2019; Berendsen et al., 2012), significantly contributes to the fitness and performance of the holobiont (Fadiji et al., 2023; Singh et al., 2020). Soils, considered as the foundation of the ‘One Health’ concept, harbor the most diverse and complex microbiomes within terrestrial ecosystems (Banerjee and van der Heijden, 2023). The rhizosphere, a narrow zone of soil influenced by root secretions, plays a crucial role in plant health. The rhizosphere microbiome acts as a primary line of defense for plants against pathogens (Banerjee and van der Heijden, 2023), and numerous microbial strains have been identified as antagonists of RKN through a range of direct and indirect interactions, including antagonism and disease suppression (Antil et al., 2023; Forghani and Hajihassani, 2020). When faced with pathogen, healthy resistant plant varieties recruit and receive a protective soil microbiome subset in the rhizosphere. This subset suppresses pathogens by competing nutrients (Du et al., 2022), producing antimicrobial substances (Luo et al., 2022), regulating plant growth (Xiang et al., 2018), and inducing plant defensive reactions (Antil et al., 2023). Previous studies have proven the potential of the soil microbiome to suppress plant-parasitic nematodes (Adam et al., 2014; Liu et al., 2020; Zhou et al., 2018), highlighting that variations in rhizospheric microbial communities can explain, to some extent, the variable infestation success of RKNs (Elhadyl et al., 2017; Lu et al., 2014). Numerous rhizospheric bacteria and fungi, such as *Bacillus* spp. (Bui et al., 2020; Liu et al., 2020), *Pseudomonas* spp. (Liu et al., 2020), *Microbacterium* spp. (Zhao J. et al., 2019; Zhao Z. B. et al., 2019), *Paecilomyces* spp. (Kiriga et al., 2018), and *Trichoderma* spp. (Khan et al., 2020; Martínez-Medina et al., 2017), have been reported to effectively control RKNs. Therefore, the rhizosphere represents one of the most promising and valuable sources of biocontrol microbiomes that remains to be explored and utilized for RKNs management.

Plant genotypes are involved in the assembly process of the rhizosphere microbiome, primarily in the form of diverse plant-derived compounds (Oyserman et al., 2022; Sasse et al., 2018), and significantly contribute to the development of specific microbiome-associated disease resistance (Lee et al., 2021; William et al., 2018). Variations in the severity of RKN diseases among different tobacco varieties in the same field suggest the presence of a specific composition and interaction of the microbiome in the rhizosphere, which plays a regulatory role in RKN. Although studies have revealed the role of rhizospheric microbes in antagonistic interactions with RKNs in great detail (Antil et al., 2023; Zhou et al., 2019), efforts to identify the core microbiome interacting with RKNs among resistant and susceptible plants in the field are still limited.

In recent years, culture-independent high-throughput sequencing has greatly expanded the repertoire of the plant-associated microbiome and its role in disease-suppression (Liu et al., 2016; Zhang S. et al., 2020). This advancement has enabled us to unravel complex microbial communities, discover novel microbial indicators, and investigate the interactions between the core microbiota and RKN in the plant rhizosphere. We hypothesized that the bacterial–fungal community in the rhizosphere contributed to tobacco’s resistance to RKN and specific microbiomes played critical antagonistic roles in manage root-knot nematodes. The aims of this study were to (1) characterize the bacterial and fungal communities in the rhizosphere of tobacco varieties that resistant and susceptible to RKN, and (2) identify bacteria and fungi potentially interact with RKN and explore potential antagonists for RKN management. The results of this study provide promising avenues for innovative RKN management strategies.

## 2 Materials and methods

### 2.1 Field experimental design

The field experiment (26°17′38″N, 102°1′10″E, elevation: 1,892 m) was conducted from May 1st to August 30th, 2021, in Liangshan Yi Autonomous Prefecture, Sichuan Province (Supplementary Figure S1). The experimental field had been subjected to continuous tobacco cropping for years and was induced with root-knot nematode (RKN) disease. The field experiment was repeated at the same location in 2022 (Supplementary Figure S2). The RKN infecting tobacco was identified as *Meloidogyne incongnita* in our previous study (Jiang et al., 2021).

Four representative tobacco varieties, Zhongchuan208 (ZC208), Yunyan87 (YY87), Yunyan85 (YY85), and Honghuadajinyuan (HD), were used in the field experiment. Tobacco plants were bred and cultured under identical conditions by the Liangshan Prefecture Branch of Sichuan Tobacco Corporation. The experimental field was divided evenly into 12 plots, each variety of tobacco was planted in 3 randomly arranged plots (each plot size was 4.8 m × 14 m), and each plot had 100 plants (4 lines × 25 plants/line, the line spacing is 1.2 m, the spacing between two close plants in one line is 0.55 m).

### 2.2 Disease investigation

To evaluate the progression of RKN disease in different tobacco varieties, the disease grades of the aboveground and root parts of each plant were investigated (Fan et al., 2023). The criteria for disease grade refer to the national standard of the tobacco pest classification survey method of China (GBT-23222-2008, Table 1). The aboveground disease grade for each plant was recorded every 20 days within 80 days after transplanting. The criteria ranked the degree of aboveground disease as grades 0 to 9 depending on plant growth and symptoms on leaves. Plants were uprooted with the whole root ball, the soil around the roots was carefully removed under running tap water 100 days after transplanting, and the root disease grade for each root was assessed. The criteria ranked the degree of root disease as grades 0 to 9 depending on the proportion of root knots that infected the root.

**Table 1:** The criteria for tobacco RKN disease grade.

**The aboveground disease grades: description of tobacco growth and leaf wilting**	
Grade 0	Normal growth
Grade l	Normal growth; the margin or tip of leaves are chlorotic but not wilted
Grade 3	One-fourth to one-third shorter than normal plants; the margin or tip of a small number of leaves are wilted
Grade 5	One-third to one-half shorter than normal plants; more than half of the leaves have wilted margins and tips or wilted spots
Grade 7	Less than half the height of normal plants; the margin and tip of all leaves are wilted, or all leaves have wilted spots
Grade 9	Plant growth is severely stunted; all leaves are wilted
**The root disease grades: description of root and root knot (s)**	
Grade 0	Normal root
Grade l	A small number of root knots on less than one-quarter of the root
Grade 3	A small number of root knots on one-quarter to one-third of the roots
Grade 5	One-third to one-half of the roots have a root knot
Grade 7	More than half of the roots have a root knot, including a small number of secondary roots
Grade 9	All roots, including secondary roots, are covered with root knots

To evaluate the difference in the degree of RKN disease among different tobacco varieties, disease incidence rate, disease index and area under the disease progression curve (AUDPC) were calculated using the following formulas:


$$\mathrm{Disease incidence rate}=\;\frac{N_{d}}{N}\times 100\%\text{.}$$

where *N_d_* is the number of disease-infected plants, and *N* is the total number of investigated plants (Ali et al., 2022).


$$\mathrm{Disease index}=\sum \frac{N_{i}\times v_{i}}{N\times 9}\times 100$$

where *N_i_* is the number of plants with the respective disease grade, *v_i_* is the disease grade (0, 1, 3, 5, 7, and 9), and *N* is the total number of investigated plants (Ali et al., 2022).


$$\mathrm{AUDPC}=\sum \left(\frac{\left(V_{i}+V_{i-1}\right)}{2}\times \left(t_{i}-t_{i-1}\right)\right)$$

where *V_i_* and *V*_*i*−1_ are the disease indices on *t_i_* (20, 40, 60, 80, 100) and *t*_*i*−1_ (0, 20, 40, 60, 80, 100), respectively, and *t_i_*-*t*_*i*−1_ is the number of days between *t_i_* and *t*_*i*−1_ (Dyer et al., 2022).

### 2.3 Sampling and measurement

To investigate the density of RKNs and identify the characteristics of the microbial community in the rhizosphere of different tobacco varieties, samples of the tobacco rhizosphere and bulk soil were collected 100 days after transplanting in the experimental field. Three rhizosphere soil samples for each tobacco variety were collected following the methods described in our previous study (Liu et al., 2016). Five individual rhizosphere soils from five random plants in each plot were collected and mixed into one sample. Five samples of representative bulk soil from five randomly arranged sites in the experimental field were collected, and the sampling depth was 10–20 cm. Each soil sample was separated into two even samples (Sample A and Sample B). Samples A and B were transported to Southwest University, Chongqing, for further testing within 3 days. Sample A was stored at room temperature (24°C) to determine the density of RKN. Sample B was stored at −20°C for DNA extraction.

RKN-infected tobacco roots were observed and photographed under a stereomicroscope. The density of second-stage juveniles of *Meloidogyne incongnita* (*M. incongnita* J2) in each soil sample was determined, considering that *M. incongnita* J2 is the infectious stage during which nematodes puncture the root tissues, resulting in knots or swellings (Abad et al., 2003). Hatched *M. incongnita* J2s in the soil samples were isolated according to the Baermann funnel method, and the populations were counted using a stage micrometer under a Nikon microscope (Tokyo, Japan). The density of *M. incongnita* J2 is expressed per 100 g of dry soil.

### 2.4 DNA extraction and sequencing

Microbial DNA from soil samples was extracted using the FastDNA Spin Kit (MP Biomedicals, United States kits) following the standard protocol. Microbial DNA was stored at −80°C. The amplification and purification of soil microbial DNA were conducted according to methods described in our previous study (Zhang et al., 2017). The 515 forward (5′-GTGCCAGCMGCCGCGG-3′) and 806 reverse (5′-GGACTACHVGGGTWTCTAAT-3′) primers were used to amplify the V4 region of the bacterial 16S rDNA gene. ITS1 forward (5′-CTTGGTCATTTAGAGGAAGTAA-3′) and ITS2 reverse (5′-GCTGCGTTCTTCATCGATGC-3′) were used to amplify the ITS1 region of the fungal ITS gene.

The 16S rRNA and ITS gene fragments were sequenced by Shanghai Majorbio Co., Ltd., China, using the Illumina MiSeqPE250 platform.^1^ The quality control and annotation of the raw sequencing data were conducted to generate high-quality reads, and amplicon sequence variants (ASVs) with a consistency of 100% were assigned to taxa at the phylum, class, order, family and genus levels using QIIME2.^2^ The taxonomic assignments of each bacterial and archaeal ASV were performed using the Silva138 database, and the taxonomic assignment of each fungal high-quality read was performed using the Unite8.0 database.

### 2.5 Data analysis and visualization

Microbial *α* diversity and *β* diversity analyses were performed using the free online platform of the Majorbio Cloud Platform.^3^ Specifically, the Chao1, Simpson, and Simpson evenness indices were calculated as microbial α diversity based on Faith’s phylogenetic metric at the ASV level. Specifically, Higher Chao1 index and Simpson evenness index suggested higher richness and evenness of the microbial communities respectively, additionally, higher Simpson index suggested lower diversity of the microbial communities. To determine the dissimilarity of the β diversity of the microbial communities, principal coordinate analysis (PCoA) was performed based on the Bray–Curtis distance according to the phylogenetic tree. To identify discriminative taxa between groups, linear discriminate analysis (LDA) effect size (LEfSe) was performed (Segata et al., 2011). A factorial Kruskal–Wallis sum-rank test (*α* = 0.05) was used in LEfSe to identify taxa from the phylum to genus level with significant differential abundances between categories (using all-against-all comparisons, LDA score > 2.0).

To determine the effect of the soil microbiome on RKNs in the tobacco rhizosphere, the underlying co-occurrence of bacteria, fungi and RKNs was determined through network analysis via the Molecular Ecological Network analysis pipeline (MEAN^4^) and Grephi software (Sun et al., 2023). Network analysis was performed at the ASV level (relative abundance greater than 0.01%) for the bacterial–fungal communities to ensure the accuracy of interactions, and network analysis was performed at the genus level to reduce the complexity of calculations and ensure the accuracy of taxonomic information. Data filtering was performed prior to avoiding zero values that could result in spurious correlations, and the taxa represented in 50% of the samples were retained (Zhao J. et al., 2019; Zhao Z. B. et al., 2019). To determine the direct effects of the α diversity of bacterial and fungal communities on RKNs, a structural equation model (SEM) was constructed in IBM SPSS AMOS software.

All statistical significance was assessed by ANOVA in SPSS Statistics software. To evaluate the correlation between variables, linear regression analysis was performed with SPSS statistics software. Other figures were generated in Origin software.

## 3 Results

### 3.1 Variation in resistance to root-knot nematode disease among different tobacco varieties

**Figure 1:**
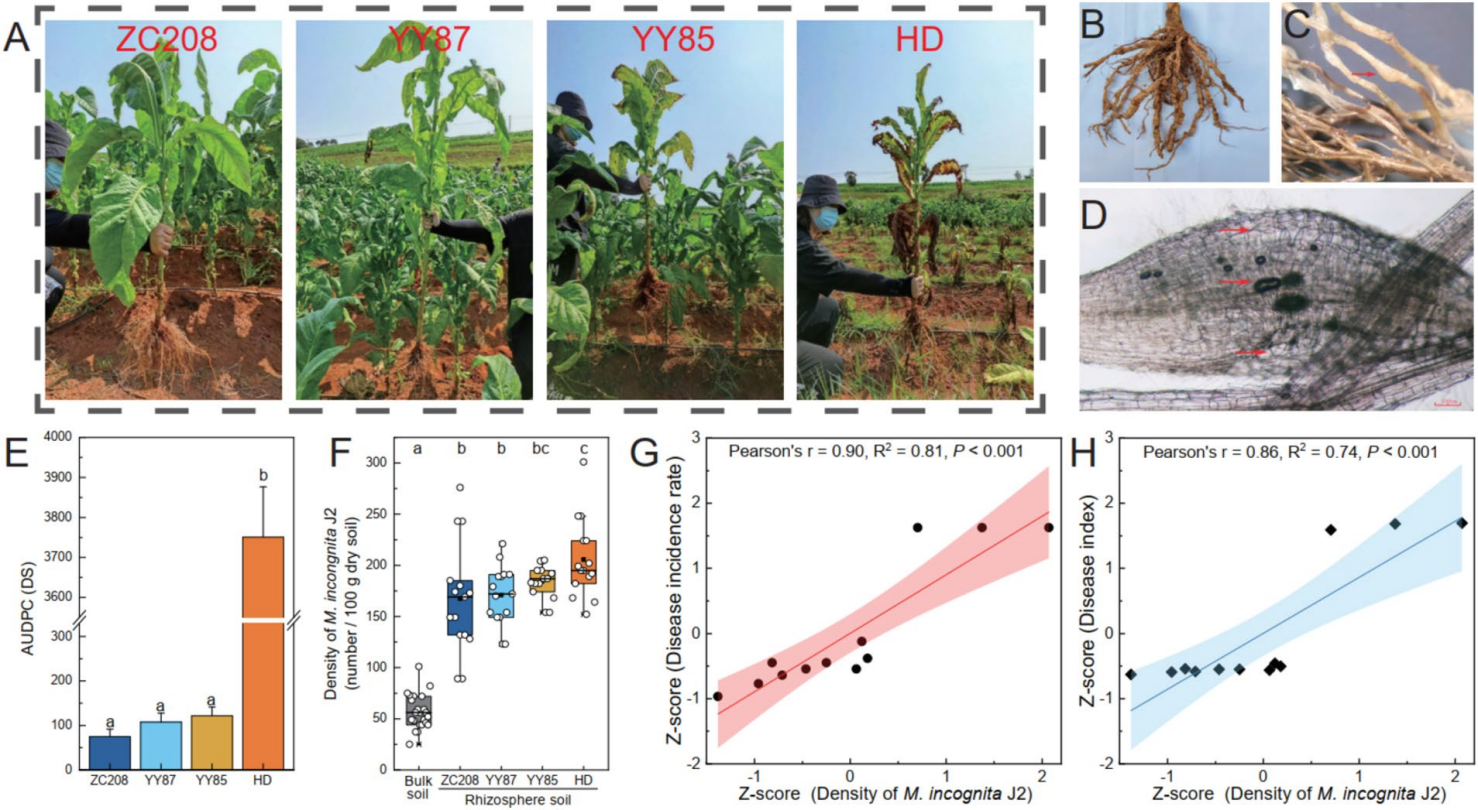
Variation in resistance to root-knot nematode disease among different tobacco varieties. (A) Disease-induced tobacco. The disease-induced tobacco roots (B) and root-knots (C). (D) The nematodes in disease-induced tobacco roots and the giant cells on the roots. (E) Area under disease progression curve (AUDPC). (F) Density of *M. incongnita* J2 populations. Relationship between disease incidence rate (G) and disease index (H) and the density of *M. incongnita* J2 populations.

The resistance levels of four different tobacco varieties to RKNs were evaluated in a field experiment (Figures 1A–D; Supplementary Figure S1). The results indicated significant differences in resistance to RKNs among the four tobacco varieties. Specifically, the resistance level of HD plants was significantly lower than that of the ZC208, YY85, and YY87 plants, whose AUDPC was 96% lower than that of the HD plants (Figure 1E). The density of *M. incongnita* J2 in both bulk soil (25 ~ 101 per 100 g dry soil) and rhizosphere soil (89 ~ 301 per 100 g dry soil) was assessed. The density of *M. incongnita* J2 in the rhizosphere was 195% greater than that in the bulk soil (*p* < 0.05), suggesting an enrichment of *M. incongnita* J2 in the tobacco rhizosphere (Figure 1F). Notably, the proportion of *M. incongnita* J2 in the rhizosphere of the HD treatment group was 11 ~ 22% greater than that in the rhizosphere of the other treatment groups. Furthermore, the density of *M. incongnita* J2 was positively correlated with the incidence rate (Figure 1G, *r* = 0.90, *p* < 0.001) and disease index (Figure 1F, *r* = 0.85, *p* < 0.001), confirming that *M. incongnita* J2 in the tobacco rhizosphere contributes positively to RKN disease outbreaks. These results suggested that HD exhibited stronger attraction to *M. incongnita* J2 and lower resistance against RKNs than ZC208, YY85, and YY87. Thus, HD was identified as a susceptible variety, while ZC208, YY85, and YY87 were categorized as resistant varieties in the following analysis.

### 3.2 Effect of the α diversity of the soil microbial community on root-knot nematodes

To reveal the effects of the soil microbiome on root-knot nematodes, the bacterial and fungal communities of the bulk soil and tobacco rhizosphere were characterized by high-throughput sequencing. For the bacterial communities, a total of 4,472,837 reads with an average read length of 256 bp were obtained through 16S rRNA high-throughput sequencing analysis. These reads were clustered into 37,313 ASVs, which were assigned to 45 bacterial phyla and six archaeal phyla. For fungal communities, 4,739,497 reads with an average read length of 241 bp were detected through ITS high-throughput sequencing analysis. These reads were clustered into 5,611 ASVs, which were assigned to five fungal phyla.

To evaluate the alpha diversity of the bacterial and fungal communities among the different sample groups, the Chao1 index, Simpson diversity index and Simpson evenness index were calculated to assess the richness, diversity and evenness of the microbial communities (Table 2). The results showed that the bacterial richness and diversity in the tobacco rhizosphere were greater than those in the bulk soil. Conversely, the richness and diversity of fungi in the rhizosphere soil were lower than those in the bulk soil, although these differences were statistical insignificance (*p* > 0.05). Our findings suggested that tobacco roots may possess an active effect on soil bacteria in the rhizosphere. Specifically, soil bacteria were generally recruited and enriched in the rhizosphere based on its higher alpha diversity of bacterial community. However, the rhizosphere effect on soil fungi was negative, and only specific fungi were selected and prosper in the tobacco rhizosphere based on its lower alpha diversity of fugal community.

**Table 2:** α diversity of the bacterial and fungal communities of the soil samples.

Microbiome type	Soil types	Chao1 richness index	Simpson diversity index	Simpson evenness index
Bacteria	Bulk soil	4423.00 ± 100.50a	0.0046 ± 0.0008a	0.0541 ± 0.0071a
	ZC208	4925.33 ± 61.76a	0.0035 ± 0.0006a	0.0615 ± 0.0093a
	YY87	4549.33 ± 423.02a	0.0036 ± 0.0007a	0.0647 ± 0.0061a
	YY85	5255.33 ± 125.06a	0.0028 ± 0.0002a	0.0687 ± 0.0030a
	HD	5014.67 ± 276.05a	0.0029 ± 0.0002a	0.0703 ± 0.0025a
Fungi	Bulk soil	711.40 ± 44.78a	0.0442 ± 0.0056a	0.0336 ± 0.0028a
	ZC208	666.67 ± 74.19a	0.1450 ± 0.0728a	0.0156 ± 0.0056a
	YY87	616.33 ± 42.81a	0.0575 ± 0.0178a	0.0329 ± 0.0075a
	YY85	659.33 ± 120.66a	0.2153 ± 0.1760a	0.0248 ± 0.0112a
	HD	671.67 ± 59.88a	0.0941 ± 0.0493a	0.0240 ± 0.0075a

A higher Chao1 richness index indicates greater richness, a higher Simpson index indicates less diversity, and a higher Simpson evenness indicates greater evenness.

**Figure 2:**
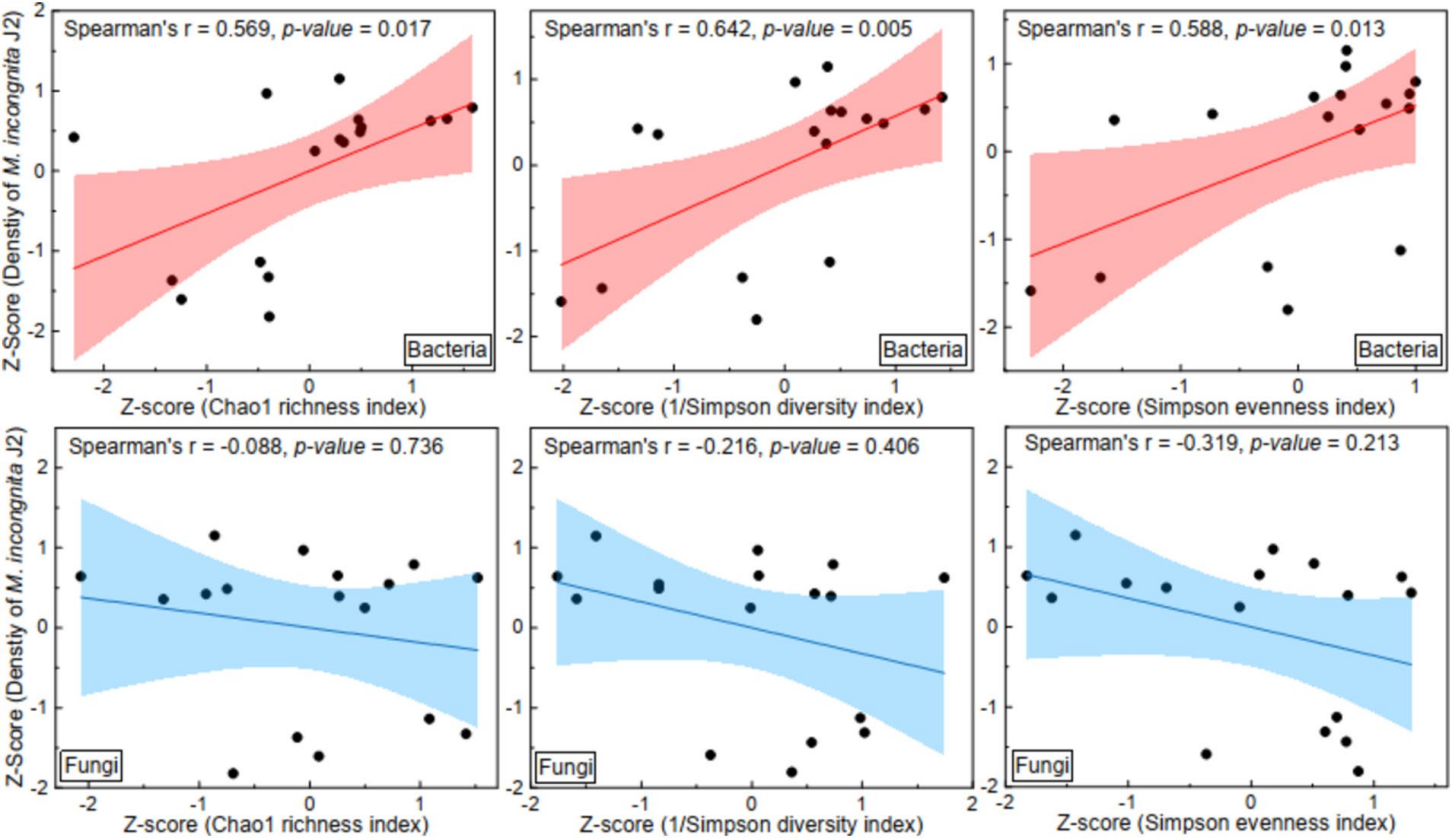
Effect of the *α* diversity of the soil microbiome on the density of *M. incongnita* J2.

Furthermore, to explore the effect of the *α* diversity of microbial communities on root-knot nematodes, linear regression analysis between the α diversity of microbial communities and the density of *M. incongnita* J2 populations was conducted; the independent variables were the Chao1 index, Simpson diversity index and Simpson evenness index, and the dependent variable was the density of *M. incongnita* J2 populations (Figure 2). The results indicated that the Chao1 index (Spearman’s *r* = 0.57, *p* < 0.05), 1/Simpson diversity index (Spearman’s *r* = 0.64, *p* < 0.05) and Simpson evenness index (Spearman’s *r* = 0.59, *p* < 0.05) of the bacterial community were positively associated with the density of *M. incongnita* J2. In contrast, the Chao1 index (Spearman’s *r* = −0.09, *p* > 0.05), 1/Simpson diversity index (Spearman’s *r* = −0.21, *p* > 0.05) and Simpson evenness index (Spearman’s *r* = −0.32, *p* > 0.05) of the fungal community showed a negative relationship with the density of *M. incongnita* J2, although these correlations did not reach statistical significance. The results suggested that the effects of soil bacterial and fungal communities on RKN were different and that the soil bacterial community had a stronger and more direct effect on RKN.

### 3.3 Differences in microbial community composition among different tobacco varieties

**Figure 3:**
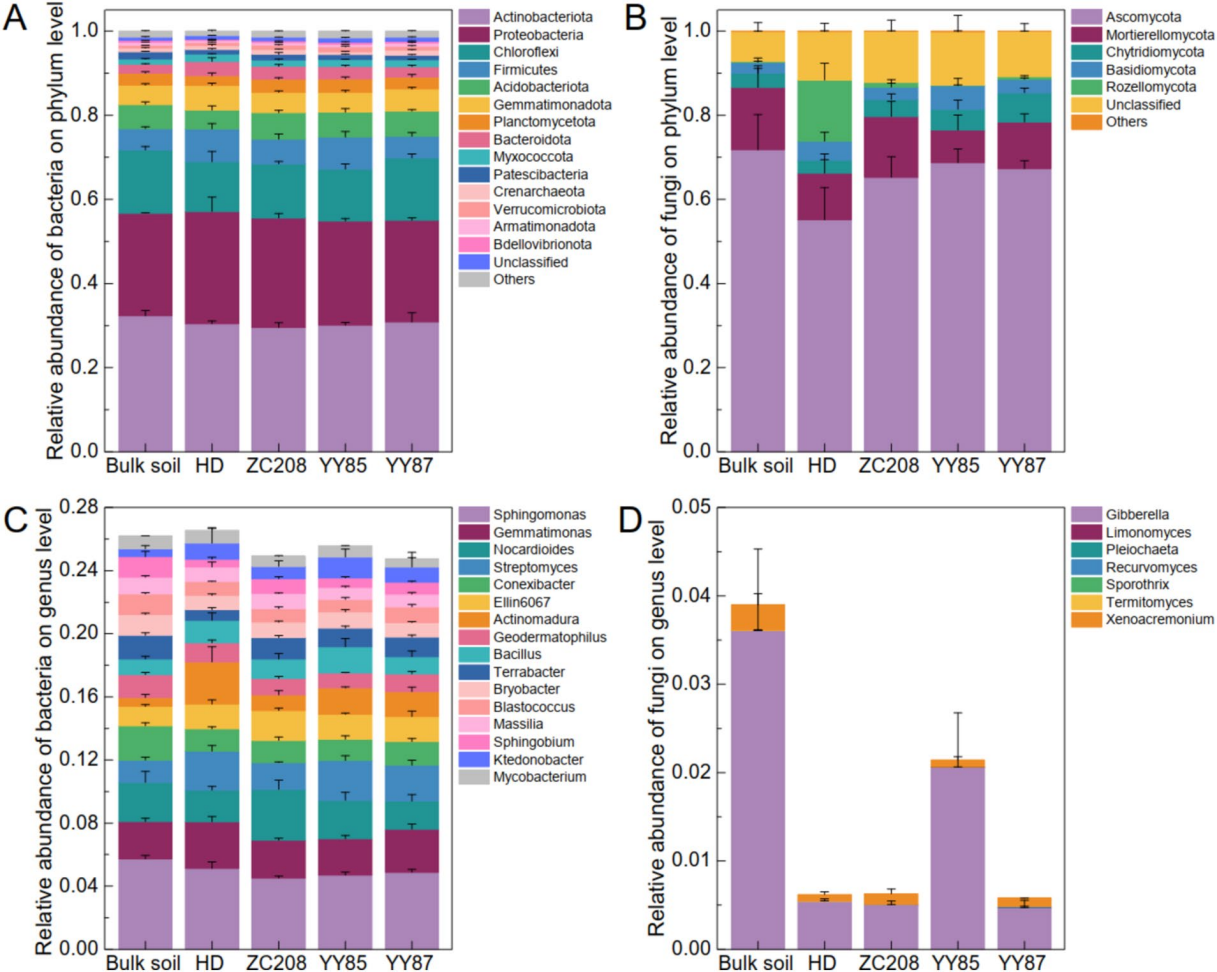
Soil microbial community composition. (A) Bacterial community composition at the phylum. (B) Fungal community composition at the phylum. The dominated genuses of bacterial (C) and fungal (D) community.

To clarify the effect of the rhizosphere on the soil microbial community, the bacterial–fungal community compositions of the bulk soil and rhizosphere of different tobacco varieties were characterized. The results suggested that the bacterial community compositions at the phylum level among the different groups were similar. Specifically, over 43% of the tested soil microbiomes were bacteria, and over 52% were fungi (Supplementary Figure S1). Actinobacteriota, Proteobacteria and Chloroflexi were the most abundant bacterial phylum, and their total relative abundances (RAs) were greater than 65% (Figure 3A). The fungal community composition at the phylum level differed significantly among the different groups. Ascomycota was the most abundant fungal phylum, whose RAs were greater than 50%. Additionally, the RAs of Ascomycota in the rhizosphere of the HD were lower than those in the bulk soil and rhizosphere of the resistant plants (Figure 3B). *Sphingomonas*, *Gemmatimonas*, *Nocardioides*, *Streptomyces*, and *Conexibacter* were the most abundant bacterial genus, remarkably, the RA of *Nocardioides* in the rhizosphere of the ZC208 were 60.85, 31.97, and 80.83% higher than those in rhizosphere of HD, YY85, and YY87, respectively (Figure 3C). Additionally, *Gibberella* and *Xenoacremonium* were the most abundant fungal genus and the RA of *Gibberella* in the bulk soil were much higher than that in the rhizosphere (Figure 3D).

**Figure 4:**
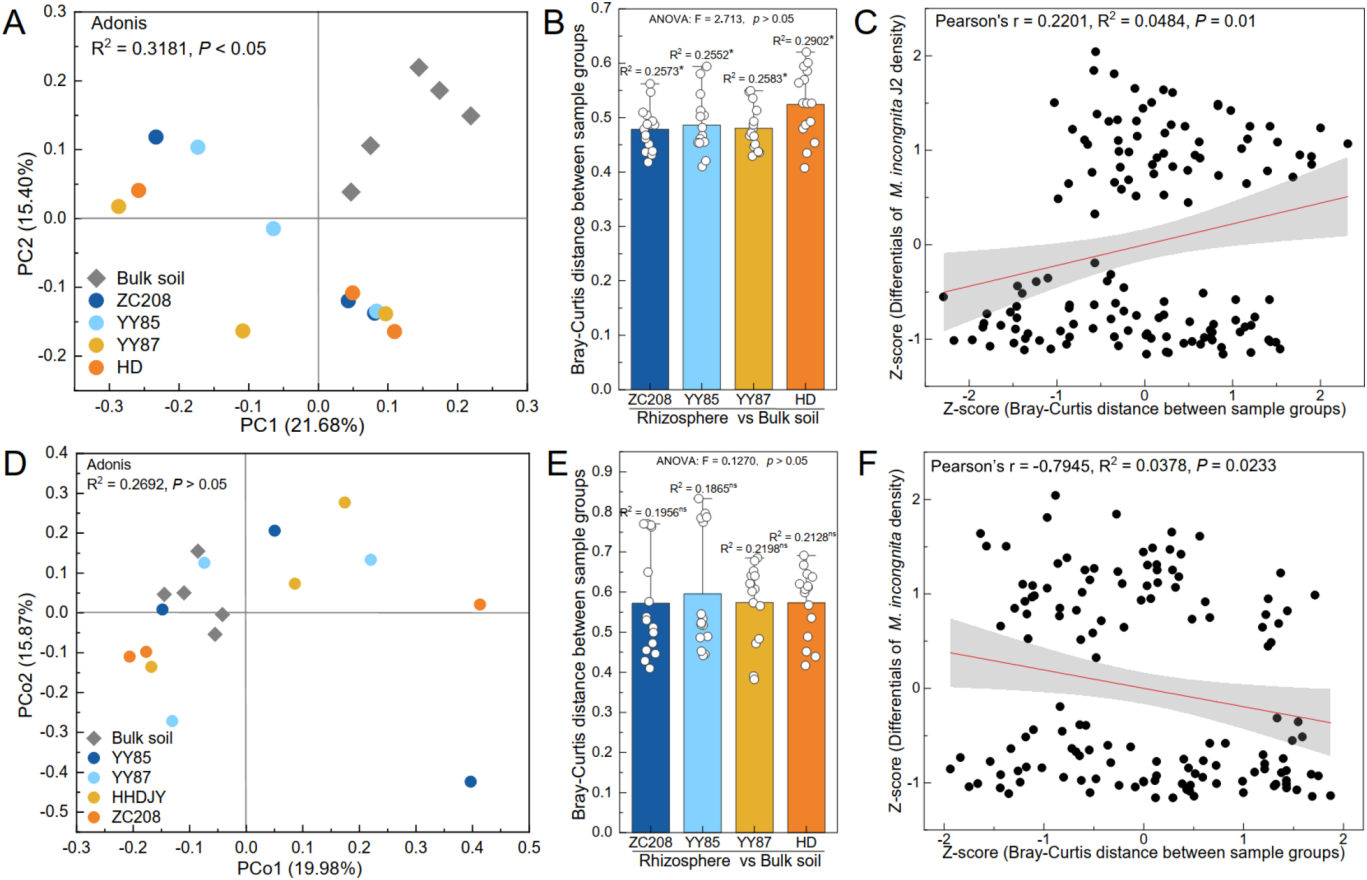
Effect of bacterial and fungal communities on the density of *M. incongnita* J2. Principal coordinate analysis (PCoA) and permutational multivariate analysis of variance (PERMANOVA) of the bacterial (A) and fungal (D) communities of the samples at the ASV level. Bray–Curtis distance of bacterial (B) and fungal (E) communities between the bulk soil and rhizosphere of the susceptible variety (HD) and resistant variety (ZC208, YY85, and YY87). The independent variables of the linear regression analyses were the Bray–Curtis distances of the bacterial (C) and fungal (F) communities, and the dependent variable was the difference in *M. incongnita* J2 density.

The variations in the microbial communities were analyzed using principal coordinate analysis (PCoA). The results showed a remarkable divergence in the soil bacterial communities between the bulk soil and rhizosphere (Figures 4A,B, Adonis, *R*^2^ = 0.32, *p* < 0.05). However, the soil fungal communities of the soil samples were not significantly different between the bulk soil and rhizosphere (Figures 4D,E, Adonis, *R*^2^ = 0.27, *p* > 0.05). Additionally, the difference in the bacterial community between the rhizosphere of HD and bulk soil was more pronounced than that observed for the resistant varieties (Figure 4B, ANOVA, *p* > 0.05). However, the variation in the fungal community between the bulk soil and rhizosphere of HD was lower than that between the bulk soil and rhizosphere of the resistant variety (Figure 4E, ANOVA, *p* > 0.05).

To identify the relationship between the microbial community composition and the density of *M. incongnita* J2, linear regression analyses were conducted, and the independent variable was the Bray–Curtis distance between samples, while the dependent variable was the difference in the density of *M. incongnita* J2 between the respective samples. The differences in the bacterial communities were positively associated with differences in the density of *M. incongnita* J2 (Figure 4C, Pearson’s *r* = 0.22, *p* < 0.05). However, the differences in fungal communities were negatively associated with the difference in the density of *M. incongnita* J2 (Figure 4F, Pearson’s *r* = −0.79, *p* < 0.05). Our findings further indicated that the soil bacterial community had a more direct effect on RKN; however, the soil fungal community demonstrated an indirect interaction with RKN.

### 3.4 Distinctive microbiomes in the tobacco rhizosphere and their relationships with RKN

**Figure 5:**
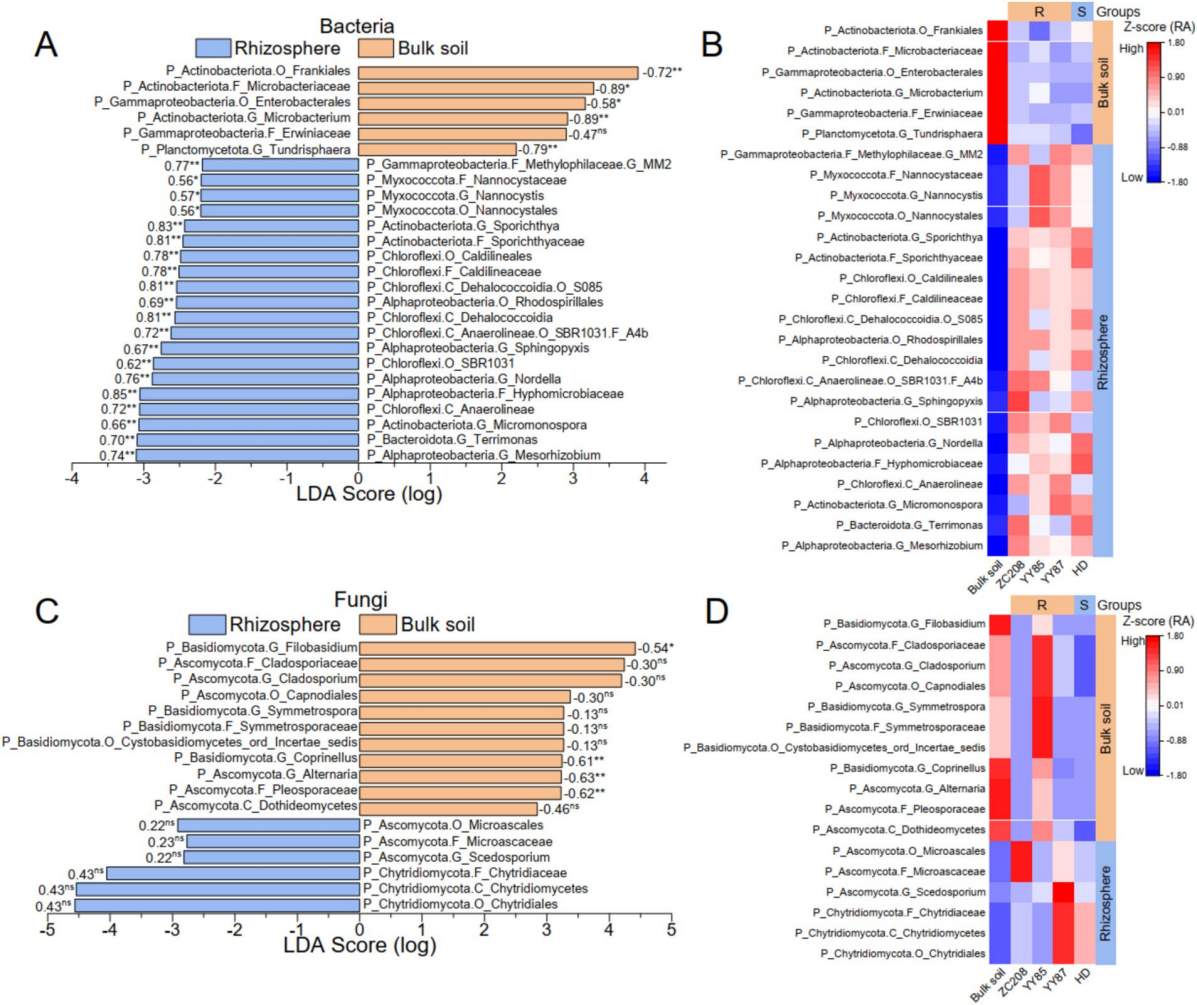
Discriminant microbial taxa associated with the density of *M. incongnita* J2. The discriminative bacterial (A) and fungal (C) taxa based on LEfSe (LDA score ≥ 2.0, *p* < 0.05) from phylum to genus between the bulk soil and the tobacco rhizosphere. Heatmaps of discriminative bacterial (B) and fungal (D) taxa based on the RA (Z score) in the rhizosphere of resistant (ZC208, YY87, and YY85) and susceptible (HD) tobacco plants. The numbers on the LDA score bars in (A,C) are Pearson’s *r* values associated with the *M. incongnita* J2 density. The *p* values from Pearson’s analysis are indicated as “ns” for *p* > 0.05, “*” for *p* < 0.05, and “**” for *p* < 0.01. The groups on the right side of the heatmaps in (B,C) highlighted the taxa enriched in the bulk soil or the rhizosphere.

To identify distinctive microbial taxa in both the tobacco rhizosphere and bulk soil, linear discriminant analysis (LDA) effect size (LEfSe) from phylum to genus was performed. Additionally, linear regression analyses between the RAs of the distinctive taxa and *M. incongnita* J2 density were conducted. Our analysis revealed 26 distinctive bacterial taxa, six of which were prevalent in bulk soil and exhibited a negative relationship with *M. incongnita* J2 density. In contrast, 20 distinctive bacterial taxa were predominant in the tobacco rhizosphere and displayed a positive correlation with *M. incongnita* J2 density (Figure 5A). Moreover, 17 distinctive fungal taxa were identified, 11 of which were more abundant in bulk soil and were negatively correlated with *M. incongnita* J2 density, while six of the identified taxa were more prevalent in the tobacco rhizosphere and were positively correlated with *M. incongnita* J2 density (Figure 5C).

To reveal the relationship between the distinctive taxa and tobacco resistance to RKNs, the RAs of distinctive bacterial (Figure 5B) and fungal (Figure 5D) taxa in resistant (ZC208, YY87, and YY85) and susceptible (HD) tobacco varieties were compared. The results showed that taxa enriched in resistant tobacco varieties, such as *Microbacterium* (Pearson’s r with J2 = −0.89, *p* < 0.01) and *Tundrisphaera* (Pearson’s r with J2 = −0.79, *p* < 0.01), were positively correlated with *M. incongnita* J2 density, and their RAs were more than 55% greater than that of HD (susceptible). Conversely, taxa prevalent in HD, such as *Terrimonas* (Pearson’s r with J2 = 0.70, *p* < 0.01), *Nordella* (Pearson’s r with J2 = 0.76, *p* < 0.01) and Hyphomicrobiaceae (Pearson’s r with J2 = 0.85, *p* < 0.01), were positively correlated with *M. incongnita* J2 density, and their RAs in the rhizosphere of HD were 21 to 53% greater than those of the resistant varieties.

### 3.5 Key microbial taxa in the bacterial–fungal co-occurrence network associated with *Meloidogyne incongnita* J2 density

**Figure 6:**
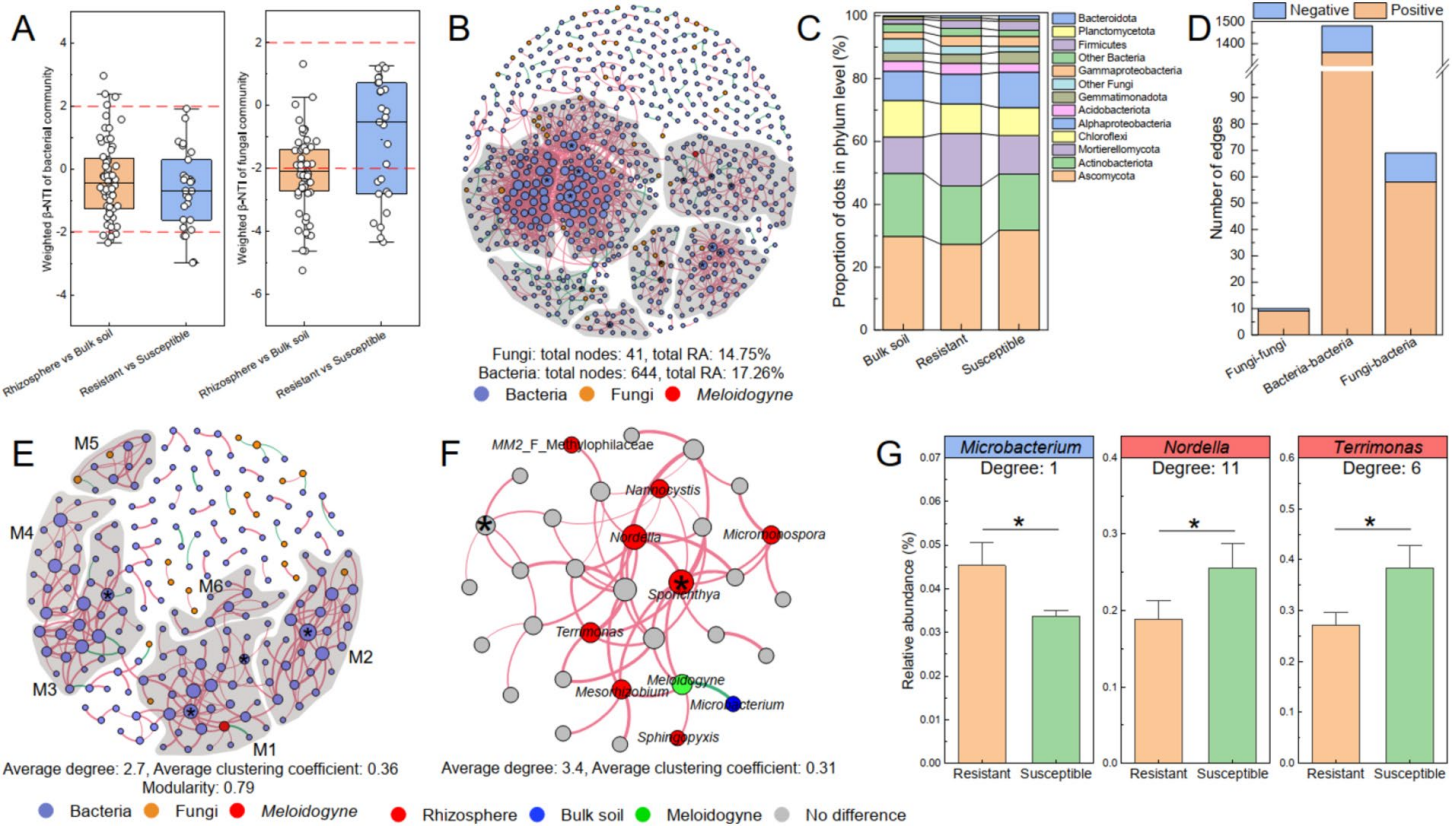
The co-occurrence network of the bacterial–fungal communities related to *Meloidogyne*. (A) *β*-NTI between sample pairs of rhizosphere and bulk soil and between resistant and susceptible varieties. The co-occurrence networks of the bacterial–fungal communities at the ASV level (B) and genus level (E). The nodes in the co-occurrence network are color coded: bacteria in blue, fungi in orange, and *M. incongnita* J2 in red. Modules were delineated with gray areas and labeled M1–M6, representing nodes with 10 or more connections. A connection between two nodes indicates a significant correlation (RA ≥ 0.01%, *r* ≥ 0.6, *p* < 0.05). Positive correlations are shown in red, whereas negative correlations are shown in green. Nodes marked with “*” represent the identified module hubs (*Zi* ≥ 2.50, *Pi* < 0.62). (C) The composition of nodes in the co-occurrence network at the phylum level. (D) The correlation of dots (ASVs) in the co-occurrence network, with distinctions between intrakingdom and interkingdom. (F) M1 of the co-occurrence network, including *M. incongnita* J2 (green), is depicted. Nodes enriched in the rhizosphere soil (red) and bulk soil (dark blue) were identified according to LEfSe. (G) Key microbial genera related to *M. incongnita* J2. Genera that were negatively correlated with J2 density are highlighted in blue boxes, while those that were positively correlated with J2 density are highlighted in red boxes. The labels indicate the degree of nodes (genus level) in module 1. * between the bars indicates significant differences at the *p* < 0.05 level.

To evaluate the assembly processes of the bacterial and fungal communities, the *β*-nearest taxon index (β-NTI) based on the null model and Sloan neutral model was calculated, and the differences in assembly processes between the rhizosphere and bulk soil, as well as between the resistant and susceptible tobacco varieties, were investigated (Figure 6A). The results revealed a predominant influence of stochastic processes (|β-NTI| < 2) in shaping the bacterial community assembly, both in the rhizosphere and bulk soil (85%) and in resistant and susceptible varieties (85%). However, for the variation in the fungal community, stochastic processes only contributed 45% of the variation between the rhizosphere and bulk soil and 63% of the variation between the resistant and susceptible varieties. These findings suggested a notable distinction between bacterial and fungal community assemblies in soil. Specifically, the fungal communities appeared to be more significantly influenced by tobacco roots than bacteria, implying a more targeted recruitment of fungal taxa by tobacco in the rhizosphere rather than a stochastic assembly.

To reveal the interaction patterns between bacterial and fungal communities, a co-occurrence network comprising 644 bacterial ASVs and 41 fungal ASVs was constructed. Within this network, the total RAs of the bacterial and fungal ASVs were 17.26 and 14.75%, respectively (Figure 6B). Notably, more than 80% of these ASVs were classified as key taxa, such as Ascomycota (29.54%), Actinobacteriota (18.86%), Mortierellomycota (13.53%), Chloroflexi (10.02%), and Alphaproteobacteria (9.96%). Among these, Mortierellomycota was more abundant in the rhizosphere of resistant tobacco than in that of susceptible tobacco (Figure 6C). Over 85% of the interactions of fungal ASVs were interkingdom (fungi–bacteria), however, bacterial ASVs engaged in interkingdom interactions were less than 5%a. Remarkably, 84.06% of interkingdom interactions were positive, suggesting that the dominant relationships between bacterial and fungal communities was symbiotic (Figure 6D). Moreover, the RAs of ASVs that participated in both interkingdom (fungi–bacteria) and intrakingdom (fungi–fungi) interactions were greater in the rhizosphere of resistant tobacco (10.60%) than in that of susceptible tobacco (8.15%), and most of the ASVs were classified as *Mortierella*, *Chaetomium*, or *Aspergillus*. This finding underscores the potential critical role of fungi in symbioticcross-kingdom interactions within the tobacco rhizosphere, but the underlying mechanism of fungal communities, such as *Mortierella*, *Chaetomium*, and *Aspergillus*, as pivotal for promoting tobacco resistance to RKNs needs further investigation.

To further identify potential keystone taxa associated with *M. incongnita* J2 density and elucidate their interaction patterns, a co-occurrence network incorporating 192 representative bacterial genera, 19 fungal genera, and *M. incongnita* J2 was established. The analysis revealed that the bacterial–fungal-J2 co-occurrence network was highly modular (modularity = 0.79), and the average degree of the network was 2.7, additionally, *M. incongnita* J2 was positioned in Modular 1 (M1, Figure 6E). To explore key genera responsive to the disease indices of J2 and RKNs, a comparative analysis was conducted between discriminative genera identified by LEfSe (distinguishing between the rhizosphere and bulk soil) and potential keystone genera within M1. Moreover, M1 was presented separately, and discriminative genera were highlighted to explore those closely related to *M. incongnita* J2 (degree = 5, Figure 6F). The results indicated that *Microbacterium,* which was significantly enriched in the bulk soil, negatively regulated *M. incongnita* J2 in the network (Spearman’s *r* = −0.89, *p* < 0.01). Conversely, *Mesorhizobium*, *Sphingopyxis*, and *Sporichthya*, which were significantly enriched in the tobacco rhizosphere, had positive interactions with *M. incongnita* J2 density. In addition, *Sporichthya* was identified as a module hub in M1 (*Zi* ≥ 2.50, *Pi* < 0.62, degree = 11), playing a crucial keystone role in maintaining the structure and function of the module. Moreover, *Nordella* (degree = 11), *Terrimonas* (degree = 6), *Nannocystis* (degree = 4), *MM2* (Methylophilaceae, degree = 1) and *Micromonospora* (degree = 4) contributed greatly to the stability and activity of M1 by widely and positively interacting with other nodes, including *M. incongnita* J2.

To clarify the differences in the distributions of key microbial taxa between resistant (ZC208, YY87, and YY85) and susceptible (HD) tobacco rhizospheres, both the discriminative taxa identified by LEfSe and key taxa associated with *M. incongnita* J2 were examined. The findings showed that the RA of *Microbacterium* was 34.49% greater in the rhizosphere of resistant tobacco than in that of susceptible tobacco (*p* < 0.05, Figure 6G). Additionally, *Nordella* and *Terrimonas* were substantially enriched in the rhizosphere of the susceptible tobacco plants by 35–41% compared with those in the rhizosphere of the resistant plants (*p* < 0.05, Figure 6G). Conversely, the RA of *Sporichthya* was only slightly greater in the rhizosphere of susceptible tobacco than in that of resistant tobacco (*p* > 0.05).

## 4 Discussion

The rhizosphere microbiome plays a critical role in determining plant health (Dastogeer et al., 2022; Yang et al., 2023), and the composition and function of microbial communities in the same soil differ between the bulk soil and rhizosphere soil and even among plant genotypes within a species (Lee et al., 2021; Oyserman et al., 2022). This study characterized the significant variations in microbial communities between the bulk soil and the rhizosphere soil of susceptible and resistant tobacco plants. Specifically, the richness and diversity of bacteria were greater in the rhizosphere soil than in the bulk soil, and the opposite trend was observed for fungi (Table 1). The results partly support that plants may actively recruit and shape their rhizosphere microbial communities, and this process is closely linked to their genotype (Berendsen et al., 2012). Similarly, Zhou et al. (2019) confirmed that noninfested rhizosphere soils from four different plants had greater microbial diversity than soils infested with RKNs, and each kind of plant hosted unique microbial communities. Furthermore, our *β*-NTI results revealed that deterministic processes predominantly govern the assembly of fungal communities in the rhizosphere, and tobacco genotype was one of the deterministic factors (Figure 6A). Additionally, our examination of the co-occurrence network of bacterial–fungal communities indicated the pivotal role of fungi, such as *Mortierella* (Borrell et al., 2017), *Chaetomium* (Spinelli et al., 2022) and *Aspergillus* (Li et al., 2023), in cross-kingdom interactions in the tobacco rhizosphere, and these fungi have been reported to play key roles in soil microecology. In conclusion, the fungal community in the tobacco rhizosphere appears to be more genotype-determined and has a substantial impact on the cross-kingdom interactions of bacterial–fungal communities. Additionally, the area of the four tobacco varieties studied was more than 80% of all tobacco-planting area in Liangshan Yi Autonomous Prefecture, Sichuan Province, and the result of variation in resistance to RKN among the four tobacco varieties were confirmed by two consecutive years of field experiment, but the generalizability of the findings in this study may be limited because of the representativeness and balance of the experimental design, since there were only one susceptible and three resistant tobacco varieties were tested. Therefore, the cross-kingdom interactions of microbial communities in the tobacco rhizosphere require further elucidation and a more balanced selection of susceptible and resistant varieties in future studies should be considered.

**Figure 7:**
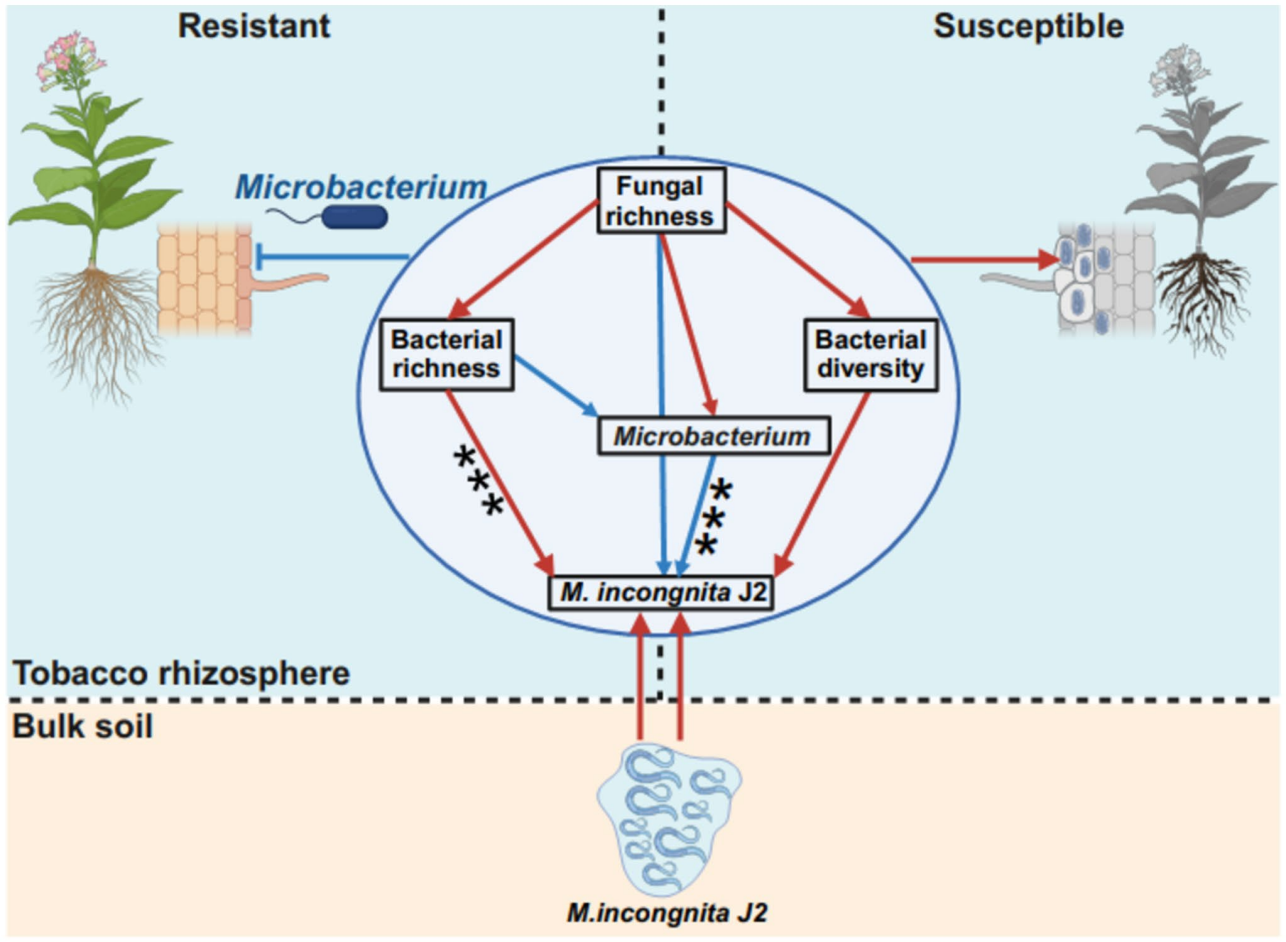
Interaction patterns of the fungal–bacterial community and *Meloidogyne*. Differences in interaction patterns between *M. incongnita* J2 and the fungal–bacterial community in the rhizosphere of resistant and susceptible tobacco plants are shown. The structural equation model (SEM) in the circle illustrates the effect of fungal richness on bacterial richness, bacterial diversity, the RA of *Microbacterium* and the density of *M. incongnita* J2. The low chi-square (χ^2^ = 0.494), nonsignificant probability level (*p* = 0.781), high goodness-of-fit index (GFI = 0.988), low Akaike information criterion (AIC = 26.494), and low root-mean-square error of approximation (RMSEA = 0.000) indicated that our data matched the hypothetical models. The red solid arrows indicate the promoting effects, and the blue solid arrows indicate the suppressing effects.

In this study, *Microbacterium* was suggested as a potential microbial antagonist against RKN and showed a significant negative correlation with *M. incongnita* J2, moreover, it enhanced presence in RKN-resistant tobacco genotypes compared to RKN-susceptible tobacco genotypes (Figure 6G). Research has demonstrated that *Microbacterium* spp. can notably improve plant resistance and suppress the penetration and development of plant-parasitic nematodes by inducing plant systemic resistance (Sahu et al., 2020; Zhao J. et al., 2019) and boosting secondary metabolites in roots (Gupta et al., 2017). Our findings further revealed that *Microbacterium* is a promising microbial antagonist for future RKN management strategies. To illustrate the interaction patterns between the fungal–bacterial community and *Meloidogyne incongnita* J2 in the tobacco rhizosphere, a model based on a structural equation model (SEM) was proposed to match the hypothetical models (Figure 7). According to this model, bacterial richness had a direct and significant positive effect on *Meloidogyne incongnita* J2 (*p* < 0.001), while *Microbacterium* had direct and significant negative effects on *Meloidogyne incongnita* J2 (*p* < 0.001). Additionally, fungal richness had direct and positive effects on bacterial richness (*p* > 0.05), bacterial diversity (*p* > 0.05) and the abundance of *Microbacterium* (*p* > 0.05). This model roughly revealed the fungi–bacteria–nematode interaction patterns in the tobacco rhizosphere for the first time, in conclusion, the bacterial community and fungal community had different effects on the interaction of RKN, specifically, the effect of the bacterial community on RKN was direct; in contrast, the effect of the fungal community on RKN was indirect which was worked through the regulation of the bacterial community. This finding confirmed both the results of linear regression analysis between the alpha diversity of microbial communities and the density of *M. incongnita* J2 (Figure 2) and the results of the occurrence network of representative ASVs in this study (Figure 6). According to these interaction patterns, the enhanced presence of *Microbacterium* in the rhizosphere of resistant tobacco possibly contributed to root resistance against RKN. Since this study identified potential microbial antagonists *Microbacterium* relying on sequencing data, the direct effect of Microbacterium on RKN suppression has not been confirmed, so the supplementary experiments demonstrating the direct effect of *Microbacterium* on RKN in the future would make this conclusion more robust and applicable to real-world scenarios. Meanwhile, whether the microbial interactions observed in this study were specific to tobacco plants or if they could be applied to other crops affected by RKN could not be evaluated and need to be further explored because of lack of similar researches.

Plant roots actively select specific microbes to thrive in the rhizosphere, which can enhance nutrient uptake and promote plant growth (Berendsen et al., 2012). In our study, various microbes that prospered in the tobacco rhizosphere (Figure 6F), such as *Nordella* (Liu et al., 2023)*, Terrimonas* (Ou et al., 2019; Wei et al., 2019; Zhang et al., 2023), *Mesorhizobium* (Hassen et al., 2020; Singh et al., 2021), *Sphingopyxis* (Verma et al., 2020), *Sporichthya* (Zhang et al., 2019), *Nannocystis* (Hüpeden et al., 2020; Sun et al., 2022), *and Micromonospora* (Martínez-Hidalgo et al., 2014; Martínez-Hidalgo et al., 2015) were identified as core members of the plant rhizosphere and endogenous microbiome. These microbes are typically involved in improving nutrient availability and promoting plant growth (Berendsen et al., 2012). As a result of the growth promotion by these special microbes, the quantity of root exudates released into the rhizosphere increases with plant growth (Bais et al., 2006), potentially explaining why microbes flourishing in the tobacco rhizosphere. Remarkably, the RAs of *Nordella* and *Terrimonas* were significantly greater in the rhizosphere of susceptible tobacco than in that of resistant tobacco (Figure 6G), suggesting a possible positive influence on RKNs. However, the specific roles of these microbes in the rhizosphere and their interactions with RKNs require further exploration to elucidate the underlying microbial drivers of RKN disease.

Second-stage juveniles (J2s) of RKNs aggregate around host root surfaces (Castagnone-Sereno et al., 2015) and subsequently invade plant roots and cause root knots (Forghani and Hajihassani, 2020). During the progression of RKN disease, the rhizosphere serves as a crucial zone for RKNs to locate and invade plant roots, hence, the density of RKNs in the rhizosphere is one of the most important factors related to disease (Topalovic and Geisen, 2023). Our findings verified that J2s significantly aggregated in the rhizosphere of tobacco plants grown in bulk soil. The density of J2s in the rhizosphere soil was 195% greater than that in the bulk soil (Figure 1). During the progression of RKNs aggregating around host root, root exudates may act as regulatory signals influencing the interactions between plant and soil microorganisms in the rhizosphere (Jing et al., 2023), and researchers had confirmed that some kinds of exudates, such as flavonoids, glucosinolates, terpenoids, and alkaloids, can stimulate and attract RKNs to the plant rhizosphere (Rasmann and Hiltpold, 2022). However, the underlying mechanism by which tobacco root exudates attract RKNs is still unknown. Remarkably, the densities of J2s in resistant (89 ~ 276 per 100 g dry soil) and susceptible (152 ~ 301 per 100 g dry soil) tobacco were similar (Figure 1F); however, the disease index was much greater in susceptible tobacco than in resistant tobacco (Figure 1E). This suggests that in addition to the density of RKNs in the rhizosphere, factors such as RKNs behavior, including RKNs activity and virulence, as well as the intrinsic characteristics of root tissues, are involved (Abad et al., 2003). The inherent characteristics of tobacco roots also contribute to tobacco RKN disease, which needs to be further clarified in the future.

## 5 Conclusion

Taken together, our study revealed that the fungal-bacterial community in the rhizosphere of tobacco are genotype-specific and may affect the microbe-associated resistance to root-knot nematodes (RKNs), and we explored the crucial roles of the fungal community in facilitating the cross-kingdom and symbiotic fungal–bacterial interactions to suppress RKNs. Moreover, *Microbacterium* is suggested as a potential and novel microbial antagonist against RKN based on its enhanced presence in RKN-resistant tobacco genotypes and the significant negative correlation with RKNs. Notably, the richness of fungal community enhanced tobacco’s microbe-associated resistance to RKN through the positive regulation of the richness and diversity of bacterial community and the relative abundance of *Microbacterium*. Our finding enhances the understanding of the interactions between RKN and soil microbiomes, provides novel insight for identifying the underlying microbial antagonists for PPN management, and highlights pathway to develop RKN biocontrol products by integrating benefit microbial communities.

## Data Availability

Raw sequencing data have been deposited into the NCBI Sequence Read Archive (SRA) database under accession number PRJNA1069245 for 16S rRNA, and PRJNA1078088 for ITS.

## Footnotes

^1^ https://cloud.majorbio.com

^2^ https://library.qiime2.org

^3^ https://www.majorbio.com

^4^ http://ieg4.rccc.ou.edu/mena/login.cgi
