# Supplementary material for: Using fungal–bacterial community analysis to explore potential microbiomes to manage Meloidogyne incongnita
Source: Front Microbiol. 2024 Oct 22;15:1415700. doi: 10.3389/fmicb.2024.1415700 (PMC11534710; doi:10.3389/fmicb.2024.1415700)
Supplement: Supplementary file 1 [file Data_Sheet_1.docx]

**
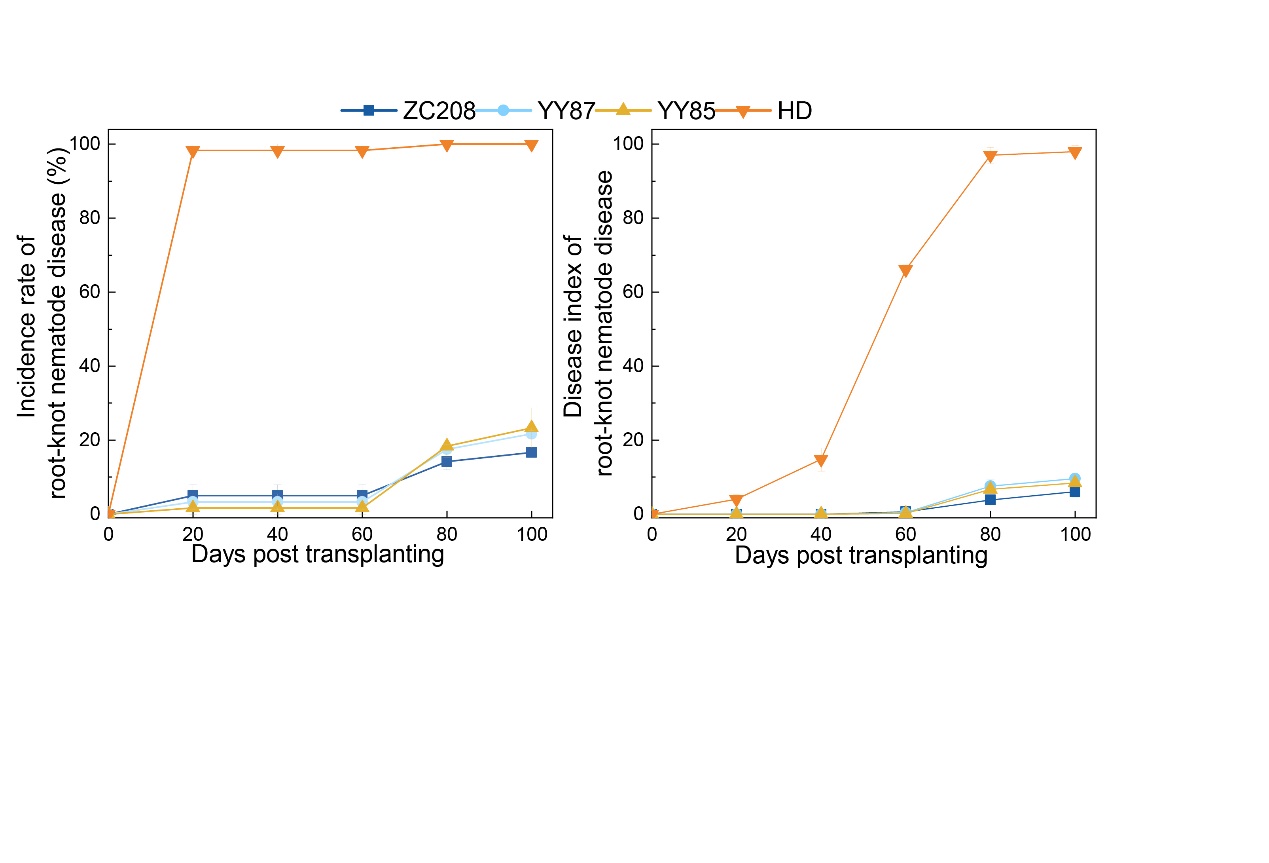
**

**Fig. S1 Resistance of different tobacco varieties to root-knot nematode disease in 2021.** Incidence rate and disease index of tobacco root-knot nematode disease within 100 d after transplanting.


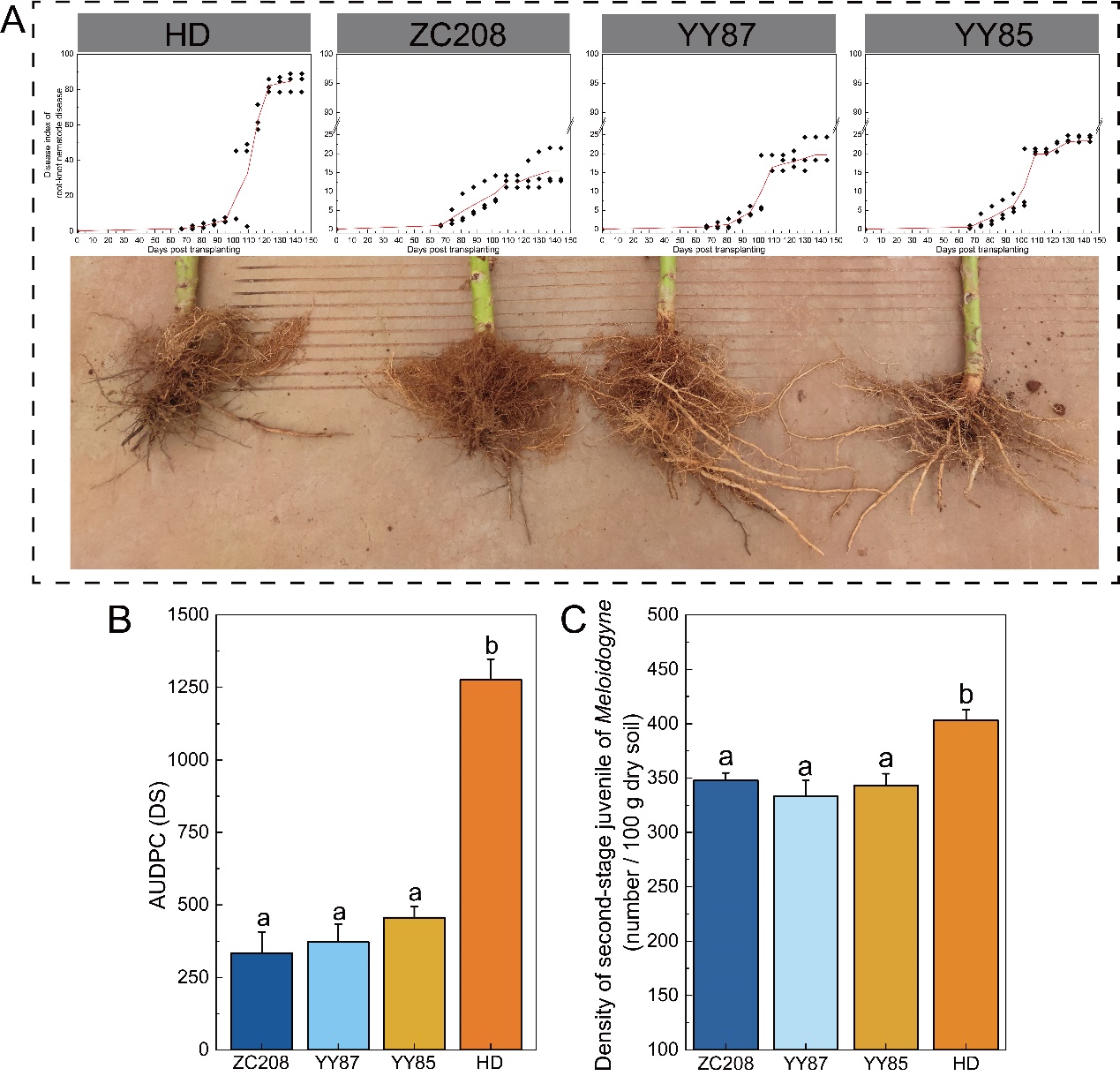


**Fig. S2 Resistance** **of different tobacco varieties to root-knot nematode disease in 2022. (A)** Disease index of tobacco root-knot nematode disease within 150 d after transplanting. Roots of different tobacco varieties 150 d after transplanting. (B) Area under disease progress curve (AUDPC) based on disease index. (C) Density of second-stage juvenile of *Meloidogyne.*
